# Reviewing the research methods literature: principles and strategies illustrated by a systematic overview of sampling in qualitative research

**DOI:** 10.1186/s13643-016-0343-0

**Published:** 2016-10-11

**Authors:** Stephen J. Gentles, Cathy Charles, David B. Nicholas, Jenny Ploeg, K. Ann McKibbon

**Affiliations:** 1 Department of Clinical Epidemiology and Biostatistics, McMaster University, Hamilton, Ontario Canada; 2 Faculty of Social Work, University of Calgary, Alberta, Canada; 3 School of Nursing, McMaster University, Hamilton, Ontario Canada; 4 CanChild Centre for Childhood Disability Research, McMaster University, 1400 Main Street West, IAHS 408, Hamilton, ON L8S 1C7 Canada

**Keywords:** Systematic review, Literature selection, Research methods, Research methodology, Overview of methods, Systematic methods overview, Review methods

## Abstract

**Background:**

Overviews of methods are potentially useful means to increase clarity and enhance collective understanding of specific methods topics that may be characterized by ambiguity, inconsistency, or a lack of comprehensiveness. This type of review represents a distinct literature synthesis method, although to date, its methodology remains relatively undeveloped despite several aspects that demand unique review procedures. The purpose of this paper is to initiate discussion about what a rigorous systematic approach to reviews of methods, referred to here as *systematic methods overviews*, might look like by providing tentative suggestions for approaching specific challenges likely to be encountered. The guidance offered here was derived from experience conducting a systematic methods overview on the topic of sampling in qualitative research.

**Results:**

The guidance is organized into several principles that highlight specific objectives for this type of review given the common challenges that must be overcome to achieve them. Optional strategies for achieving each principle are also proposed, along with discussion of how they were successfully implemented in the overview on sampling. We describe seven paired principles and strategies that address the following aspects: delimiting the initial set of publications to consider, searching beyond standard bibliographic databases, searching without the availability of relevant metadata, selecting publications on purposeful conceptual grounds, defining concepts and other information to abstract iteratively, accounting for inconsistent terminology used to describe specific methods topics, and generating rigorous verifiable analytic interpretations. Since a broad aim in systematic methods overviews is to describe and interpret the relevant literature in qualitative terms, we suggest that iterative decision making at various stages of the review process, and a rigorous qualitative approach to analysis are necessary features of this review type.

**Conclusions:**

We believe that the principles and strategies provided here will be useful to anyone choosing to undertake a systematic methods overview. This paper represents an initial effort to promote high quality critical evaluations of the literature regarding problematic methods topics, which have the potential to promote clearer, shared understandings, and accelerate advances in research methods. Further work is warranted to develop more definitive guidance.

**Electronic supplementary material:**

The online version of this article (doi:10.1186/s13643-016-0343-0) contains supplementary material, which is available to authorized users.

## Background

While reviews of methods are not new, they represent a distinct review type whose methodology remains relatively under-addressed in the literature despite the clear implications for unique review procedures. One of few examples to describe it is a chapter containing reflections of two contributing authors in a book of 21 reviews on methodological topics compiled for the British National Health Service, Health Technology Assessment Program [1]. Notable is their observation of how the differences between the methods reviews and conventional quantitative systematic reviews, specifically attributable to their varying content and purpose, have implications for defining what qualifies as systematic. While the authors describe general aspects of “systematicity” (including rigorous application of a methodical search, abstraction, and analysis), they also describe a high degree of variation within the category of methods reviews itself and so offer little in the way of concrete guidance. In this paper, we present tentative concrete guidance, in the form of a preliminary set of proposed principles and optional strategies, for a rigorous systematic approach to reviewing and evaluating the literature on quantitative or qualitative methods topics. For purposes of this article, we have used the term *systematic methods overview* to emphasize the notion of a systematic approach to such reviews.

The conventional focus of rigorous literature reviews (i.e., review types for which systematic methods have been codified, including the various approaches to quantitative systematic reviews [2–4], and the numerous forms of qualitative and mixed methods literature synthesis [5–10]) is to synthesize empirical research findings from multiple studies. By contrast, the focus of overviews of methods, including the systematic approach we advocate, is to synthesize guidance on methods topics. The literature consulted for such reviews may include the methods literature, methods-relevant sections of empirical research reports, or both. Thus, this paper adds to previous work published in this journal—namely, recent preliminary guidance for conducting reviews of theory [11]—that has extended the application of systematic review methods to novel review types that are concerned with subject matter other than empirical research findings.

Published examples of methods overviews illustrate the varying objectives they can have. One objective is to establish methodological standards for appraisal purposes. For example, reviews of existing quality appraisal standards have been used to propose universal standards for appraising the quality of primary qualitative research [12] or evaluating qualitative research reports [13]. A second objective is to survey the methods-relevant sections of empirical research reports to establish current practices on methods use and reporting practices, which Moher and colleagues [14] recommend as a means for establishing the needs to be addressed in reporting guidelines (see, for example [15, 16]). A third objective for a methods review is to offer clarity and enhance collective understanding regarding a specific methods topic that may be characterized by ambiguity, inconsistency, or a lack of comprehensiveness within the available methods literature. An example of this is a overview whose objective was to review the inconsistent definitions of intention-to-treat analysis (the methodologically preferred approach to analyze randomized controlled trial data) that have been offered in the methods literature and propose a solution for improving conceptual clarity [17]. Such reviews are warranted because students and researchers who must learn or apply research methods typically lack the time to systematically search, retrieve, review, and compare the available literature to develop a thorough and critical sense of the varied approaches regarding certain controversial or ambiguous methods topics.

While *systematic methods overviews*, as a review type, include both reviews of the methods literature and reviews of methods-relevant sections from empirical study reports, the guidance provided here is primarily applicable to reviews of the methods literature since it was derived from the experience of conducting such a review [18], described below. To our knowledge, there are no well-developed proposals on how to rigorously conduct such reviews. Such guidance would have the potential to improve the thoroughness and credibility of critical evaluations of the methods literature, which could increase their utility as a tool for generating understandings that advance research methods, both qualitative and quantitative. Our aim in this paper is thus to initiate discussion about what might constitute a rigorous approach to systematic methods overviews. While we hope to promote rigor in the conduct of systematic methods overviews wherever possible, we do not wish to suggest that all methods overviews need be conducted to the same standard. Rather, we believe that the level of rigor may need to be tailored pragmatically to the specific review objectives, which may not always justify the resource requirements of an intensive review process.

### The example systematic methods overview on sampling in qualitative research

The principles and strategies we propose in this paper are derived from experience conducting a systematic methods overview on the topic of sampling in qualitative research [18]. The main objective of that methods overview was to bring clarity and deeper understanding of the prominent concepts related to sampling in qualitative research (purposeful sampling strategies, saturation, etc.). Specifically, we interpreted the available guidance, commenting on areas lacking clarity, consistency, or comprehensiveness (without proposing any recommendations on how to do sampling). This was achieved by a comparative and critical analysis of publications representing the most influential (i.e., highly cited) guidance across several methodological traditions in qualitative research.

The specific methods and procedures for the overview on sampling [18] from which our proposals are derived were developed both after soliciting initial input from local experts in qualitative research and an expert health librarian (KAM) and through ongoing careful deliberation throughout the review process. To summarize, in that review, we employed a transparent and rigorous approach to search the methods literature, selected publications for inclusion according to a purposeful and iterative process, abstracted textual data using structured abstraction forms, and analyzed (synthesized) the data using a systematic multi-step approach featuring abstraction of text, summary of information in matrices, and analytic comparisons.

For this article, we reflected on both the problems and challenges encountered at different stages of the review and our means for selecting justifiable procedures to deal with them. Several principles were then derived by considering the generic nature of these problems, while the generalizable aspects of the procedures used to address them formed the basis of optional strategies. Further details of the specific methods and procedures used in the overview on qualitative sampling are provided below to illustrate both the types of objectives and challenges that reviewers will likely need to consider and our approach to implementing each of the principles and strategies.

### Organization of the guidance into principles and strategies

For the purposes of this article, *principles* are general statements outlining what we propose are important aims or considerations within a particular review process, given the unique objectives or challenges to be overcome with this type of review. These statements follow the general format, “considering the objective or challenge of X, we propose Y to be an important aim or consideration.” *Strategies* are optional and flexible approaches for implementing the previous principle outlined. Thus, generic challenges give rise to principles, which in turn give rise to strategies.

We organize the principles and strategies below into three sections corresponding to processes characteristic of most systematic literature synthesis approaches: *literature identification and selection*; *data abstraction* from the publications selected for inclusion; and *analysis*, including critical appraisal and synthesis of the abstracted data. Within each section, we also describe the specific methodological decisions and procedures used in the overview on sampling in qualitative research [18] to illustrate how the principles and strategies for each review process were applied and implemented in a specific case. We expect this guidance and accompanying illustrations will be useful for anyone considering engaging in a methods overview, particularly those who may be familiar with conventional systematic review methods but may not yet appreciate some of the challenges specific to reviewing the methods literature.

## Results and discussion

### Literature identification and selection

The identification and selection process includes search and retrieval of publications and the development and application of inclusion and exclusion criteria to select the publications that will be abstracted and analyzed in the final review. Literature identification and selection for overviews of the methods literature is challenging and potentially more resource-intensive than for most reviews of empirical research. This is true for several reasons that we describe below, alongside discussion of the potential solutions. Additionally, we suggest in this section how the selection procedures can be chosen to match the specific analytic approach used in methods overviews.

#### Delimiting a manageable set of publications

One aspect of methods overviews that can make identification and selection challenging is the fact that the universe of literature containing potentially relevant information regarding most methods-related topics is expansive and often unmanageably so. Reviewers are faced with two large categories of literature: the *methods literature*, where the possible publication types include journal articles, books, and book chapters; and the *methods-relevant sections of empirical study reports*, where the possible publication types include journal articles, monographs, books, theses, and conference proceedings. In our systematic overview of sampling in qualitative research, exhaustively searching (including retrieval and first-pass screening) all publication types across both categories of literature for information on a single methods-related topic was too burdensome to be feasible. The following proposed principle follows from the need to delimit a manageable set of literature for the review.

##### Principle #1:

Considering the broad universe of potentially relevant literature, we propose that an important objective early in the identification and selection stage is to delimit a manageable set of methods-relevant publications in accordance with the objectives of the methods overview.

##### Strategy #1:

To limit the set of methods-relevant publications that must be managed in the selection process, reviewers have the option to initially review only the methods literature, and exclude the methods-relevant sections of empirical study reports, provided this aligns with the review’s particular objectives.

We propose that reviewers are justified in choosing to select only the methods literature when the objective is to map out the range of recognized concepts relevant to a methods topic, to summarize the most authoritative or influential definitions or meanings for methods-related concepts, or to demonstrate a problematic lack of clarity regarding a widely established methods-related concept and potentially make recommendations for a preferred approach to the methods topic in question. For example, in the case of the methods overview on sampling [18], the primary aim was to define areas lacking in clarity for multiple widely established sampling-related topics. In the review on intention-to-treat in the context of missing outcome data [17], the authors identified a lack of clarity based on multiple inconsistent definitions in the literature and went on to recommend separating the issue of how to handle missing outcome data from the issue of whether an intention-to-treat analysis can be claimed.

In contrast to strategy #1, it may be appropriate to select the methods-relevant sections of empirical study reports when the objective is to illustrate how a methods concept is operationalized in research practice or reported by authors. For example, one could review all the publications in 2 years’ worth of issues of five high-impact field-related journals to answer questions about how researchers describe implementing a particular method or approach, or to quantify how consistently they define or report using it. Such reviews are often used to highlight gaps in the reporting practices regarding specific methods, which may be used to justify items to address in reporting guidelines (for example, [14–16]).

It is worth recognizing that other authors have advocated broader positions regarding the scope of literature to be considered in a review, expanding on our perspective. Suri [10] (who, like us, emphasizes how different sampling strategies are suitable for different literature synthesis objectives) has, for example, described a two-stage literature sampling procedure (pp. 96–97). First, reviewers use an initial approach to conduct a broad overview of the field—for reviews of methods topics, this would entail an initial review of the research *methods* literature. This is followed by a second more focused stage in which practical examples are purposefully selected—for methods reviews, this would involve sampling the *empirical* literature to illustrate key themes and variations. While this approach is seductive in its capacity to generate more in depth and interpretive analytic findings, some reviewers may consider it too resource-intensive to include the second step no matter how selective the purposeful sampling. In the overview on sampling where we stopped after the first stage [18], we discussed our selective focus on the methods literature as a limitation that left opportunities for further analysis of the literature. We explicitly recommended, for example, that theoretical sampling was a topic for which a future review of the methods sections of empirical reports was justified to answer specific questions identified in the primary review.

Ultimately, reviewers must make pragmatic decisions that balance resource considerations, combined with informed predictions about the depth and complexity of literature available on their topic, with the stated objectives of their review. The remaining principles and strategies apply primarily to overviews that include the methods literature, although some aspects may be relevant to reviews that include empirical study reports.

#### Searching beyond standard bibliographic databases

An important reality affecting identification and selection in overviews of the methods literature is the increased likelihood for relevant publications to be located in sources other than journal articles (which is usually not the case for overviews of empirical research, where journal articles generally represent the primary publication type). In the overview on sampling [18], out of 41 full-text publications retrieved and reviewed, only 4 were journal articles, while 37 were books or book chapters. Since many books and book chapters did not exist electronically, their full text had to be physically retrieved in hardcopy, while 11 publications were retrievable only through interlibrary loan or purchase request. The tasks associated with such retrieval are substantially more time-consuming than electronic retrieval. Since a substantial proportion of methods-related guidance may be located in publication types that are less comprehensively indexed in standard bibliographic databases, identification and retrieval thus become complicated processes.

##### Principle #2:

Considering that important sources of methods guidance can be located in non-journal publication types (e.g., books, book chapters) that tend to be poorly indexed in standard bibliographic databases, it is important to consider alternative search methods for identifying relevant publications to be further screened for inclusion.

##### Strategy #2:

To identify books, book chapters, and other non-journal publication types not thoroughly indexed in standard bibliographic databases, reviewers may choose to consult one or more of the following less standard sources: Google Scholar, publisher web sites, or expert opinion.

In the case of the overview on sampling in qualitative research [18], Google Scholar had two advantages over other standard bibliographic databases: it indexes and returns records of books and book chapters likely to contain guidance on qualitative research methods topics; and it has been validated as providing higher citation counts than ISI Web of Science (a producer of numerous bibliographic databases accessible through institutional subscription) for several non-biomedical disciplines including the social sciences where qualitative research methods are prominently used [19–21]. While we identified numerous useful publications by consulting experts, the author publication lists generated through Google Scholar searches were uniquely useful to identify more recent editions of methods books identified by experts.

#### Searching without relevant metadata

Determining what publications to select for inclusion in the overview on sampling [18] could only rarely be accomplished by reviewing the publication’s metadata. This was because for the many books and other non-journal type publications we identified as possibly relevant, the potential content of interest would be located in only a subsection of the publication. In this common scenario for reviews of the methods literature (as opposed to methods overviews that include empirical study reports), reviewers will often be unable to employ standard title, abstract, and keyword database searching or screening as a means for selecting publications.

##### Principle #3:

Considering that the presence of information about the topic of interest may not be indicated in the metadata for books and similar publication types, it is important to consider other means of identifying potentially useful publications for further screening.

##### Strategy #3:

One approach to identifying potentially useful books and similar publication types is to consider what classes of such publications (e.g., all methods manuals for a certain research approach) are likely to contain relevant content, then identify, retrieve, and review the full text of corresponding publications to determine whether they contain information on the topic of interest.

In the example of the overview on sampling in qualitative research [18], the topic of interest (sampling) was one of numerous topics covered in the general qualitative research methods manuals. Consequently, examples from this class of publications first had to be identified for retrieval according to non-keyword-dependent criteria. Thus, all methods manuals within the three research traditions reviewed (grounded theory, phenomenology, and case study) that might contain discussion of sampling were sought through Google Scholar and expert opinion, their full text obtained, and hand-searched for relevant content to determine eligibility. We used tables of contents and index sections of books to aid this hand searching.

#### Purposefully selecting literature on conceptual grounds

A final consideration in methods overviews relates to the type of analysis used to generate the review findings. Unlike quantitative systematic reviews where reviewers aim for accurate or unbiased quantitative estimates—something that requires identifying and selecting the literature exhaustively to obtain all relevant data available (i.e., a complete sample)—in methods overviews, reviewers must describe and interpret the relevant literature in qualitative terms to achieve review objectives. In other words, the aim in methods overviews is to seek coverage of the qualitative concepts relevant to the methods topic at hand. For example, in the overview of sampling in qualitative research [18], achieving review objectives entailed providing conceptual coverage of eight sampling-related topics that emerged as key domains. The following principle recognizes that literature sampling should therefore support generating qualitative conceptual data as the input to analysis.

##### Principle #4:

Since the analytic findings of a systematic methods overview are generated through qualitative description and interpretation of the literature on a specified topic, selection of the literature should be guided by a purposeful strategy designed to achieve adequate conceptual coverage (i.e., representing an appropriate degree of variation in relevant ideas) of the topic according to objectives of the review.

##### Strategy #4:

One strategy for choosing the purposeful approach to use in selecting the literature according to the review objectives is to consider whether those objectives imply exploring concepts either at a broad overview level, in which case combining maximum variation selection with a strategy that limits yield (e.g., critical case, politically important, or sampling for influence—described below) may be appropriate; or in depth, in which case purposeful approaches aimed at revealing innovative cases will likely be necessary.

In the methods overview on sampling, the implied scope was broad since we set out to review publications on sampling across three divergent qualitative research traditions—grounded theory, phenomenology, and case study—to facilitate making informative conceptual comparisons. Such an approach would be analogous to maximum variation sampling.

At the same time, the purpose of that review was to critically interrogate the clarity, consistency, and comprehensiveness of literature from these traditions that was “most likely to have widely influenced students’ and researchers’ ideas about sampling” (p. 1774) [18]. In other words, we explicitly set out to review and critique the most established and influential (and therefore dominant) literature, since this represents a common basis of knowledge among students and researchers seeking understanding or practical guidance on sampling in qualitative research. To achieve this objective, we purposefully sampled publications according to the criterion of *influence*, which we operationalized as how often an author or publication has been referenced in print or informal discourse. This second sampling approach also limited the literature we needed to consider within our broad scope review to a manageable amount.

**Fig. 1:**
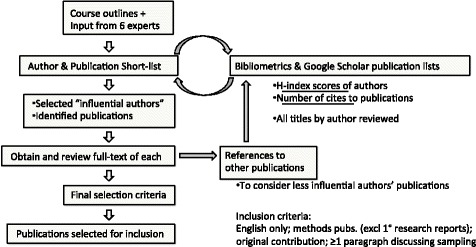
Literature identification and selection process used in the methods overview on sampling [18]

To operationalize this strategy of *sampling for influence*, we sought to identify both the most influential authors within a qualitative research tradition (all of whose citations were subsequently screened) and the most influential publications on the topic of interest by non-influential authors. This involved a flexible approach that combined multiple indicators of *influence* to avoid the dilemma that any single indicator might provide inadequate coverage. These indicators included bibliometric data (h-index for author influence [22]; number of cites for publication influence), expert opinion, and cross-references in the literature (i.e., snowball sampling). As a final selection criterion, a publication was included only if it made an original contribution in terms of novel guidance regarding sampling or a related concept; thus, purely secondary sources were excluded. Publish or Perish software (Anne-Wil Harzing; available at http://www.harzing.com/resources/publish-or-perish) was used to generate bibliometric data via the Google Scholar database. Figure 1 illustrates how identification and selection in the methods overview on sampling was a multi-faceted and iterative process. The authors selected as influential, and the publications selected for inclusion or exclusion are listed in Additional file 1 (Matrices 1, 2a, 2b).

In summary, the strategies of seeking maximum variation and sampling for influence were employed in the sampling overview to meet the specific review objectives described. Reviewers will need to consider the full range of purposeful literature sampling approaches at their disposal in deciding what best matches the specific aims of their own reviews. Suri [10] has recently retooled Patton’s well-known typology of purposeful sampling strategies (originally intended for primary research) for application to literature synthesis, providing a useful resource in this respect.

### Data abstraction

The purpose of data abstraction in rigorous literature reviews is to locate and record all data relevant to the topic of interest from the full text of included publications, making them available for subsequent analysis. Conventionally, a data abstraction form—consisting of numerous distinct conceptually defined fields to which corresponding information from the source publication is recorded—is developed and employed. There are several challenges, however, to the processes of developing the abstraction form and abstracting the data itself when conducting methods overviews, which we address here. Some of these problems and their solutions may be familiar to those who have conducted qualitative literature syntheses, which are similarly conceptual.

#### Iteratively defining conceptual information to abstract

In the overview on sampling [18], while we surveyed multiple sources beforehand to develop a list of concepts relevant for abstraction (e.g., purposeful sampling strategies, saturation, sample size), there was no way for us to anticipate some concepts prior to encountering them in the review process. Indeed, in many cases, reviewers are unable to determine the complete set of methods-related concepts that will be the focus of the final review a priori without having systematically reviewed the publications to be included. Thus, defining what information to abstract beforehand may not be feasible.

##### Principle #5:

Considering the potential impracticality of defining a complete set of relevant methods-related concepts from a body of literature one has not yet systematically read, selecting and defining fields for data abstraction must often be undertaken iteratively. Thus, concepts to be abstracted can be expected to grow and change as data abstraction proceeds.

##### Strategy #5:

Reviewers can develop an initial form or set of concepts for abstraction purposes according to standard methods (e.g., incorporating expert feedback, pilot testing) and remain attentive to the need to iteratively revise it as concepts are added or modified during the review. Reviewers should document revisions and return to re-abstract data from previously abstracted publications as the new data requirements are determined.

In the sampling overview [18], we developed and maintained the abstraction form in Microsoft Word. We derived the initial set of abstraction fields from our own knowledge of relevant sampling-related concepts, consultation with local experts, and reviewing a pilot sample of publications. Since the publications in this review included a large proportion of books, the abstraction process often began by flagging the broad sections within a publication containing topic-relevant information for detailed review to identify text to abstract. When reviewing flagged text, the reviewer occasionally encountered an unanticipated concept significant enough to warrant being added as a new field to the abstraction form. For example, a field was added to capture how authors described the timing of sampling decisions, whether before (a priori) or after (ongoing) starting data collection, or whether this was unclear. In these cases, we systematically documented the modification to the form and returned to previously abstracted publications to abstract any information that might be relevant to the new field.

The logic of this strategy is analogous to the logic used in a form of research synthesis called *best fit framework synthesis* (BFFS) [23–25]. In that method, reviewers initially code evidence using an a priori framework they have selected. When evidence cannot be accommodated by the selected framework, reviewers then develop new themes or concepts from which they construct a new expanded framework. Both the strategy proposed and the BFFS approach to research synthesis are notable for their rigorous and transparent means to adapt a final set of concepts to the content under review.

#### Accounting for inconsistent terminology

An important complication affecting the abstraction process in methods overviews is that the language used by authors to describe methods-related concepts can easily vary across publications. For example, authors from different qualitative research traditions often use different terms for similar methods-related concepts. Furthermore, as we found in the sampling overview [18], there may be cases where no identifiable term, phrase, or label for a methods-related concept is used at all, and a description of it is given instead. This can make searching the text for relevant concepts based on keywords unreliable.

##### Principle #6:

Since accepted terms may not be used consistently to refer to methods concepts, it is necessary to rely on the definitions for concepts, rather than keywords, to identify relevant information in the publication to abstract.

##### Strategy #6:

An effective means to systematically identify relevant information is to develop and iteratively adjust written definitions for key concepts (corresponding to abstraction fields) that are consistent with and as inclusive of as much of the literature reviewed as possible. Reviewers then seek information that matches these definitions (rather than keywords) when scanning a publication for relevant data to abstract.

In the abstraction process for the sampling overview [18], we noted the several concepts of interest to the review for which abstraction by keyword was particularly problematic due to inconsistent terminology across publications: *sampling*, *purposeful sampling*, *sampling strategy*, and *saturation* (for examples, see Additional file 1, Matrices 3a, 3b, 4). We iteratively developed definitions for these concepts by abstracting text from publications that either provided an explicit definition or from which an implicit definition could be derived, which was recorded in fields dedicated to the concept’s definition. Using a method of constant comparison, we used text from definition fields to inform and modify a centrally maintained definition of the corresponding concept to optimize its fit and inclusiveness with the literature reviewed. Table 1 shows, as an example, the final definition constructed in this way for one of the central concepts of the review, qualitative *sampling*.

**Table 1:** Final definition for qualitative *sampling*, including methodological tradition-specific variations

Term	Definition and tradition-specific variations
*Sampling*	The selection of specific data sources from which data are collected in order to address the research objectives
In grounded theory	What is selected (i.e., the sampling unit) in theoretical sampling is unclear or inconsistent between authors (i.e., it may not simply be data sources)
In phenomenology	What is selected is restricted to people only (i.e., a single type of data source)
In case study	What is selected includes cases (i.e., in addition to data sources)

Developed after numerous iterations in the methods overview on sampling [18]

We applied iteratively developed definitions when making decisions about what specific text to abstract for an existing field, which allowed us to abstract concept-relevant data even if no recognized keyword was used. For example, this was the case for the sampling-related concept, *saturation*, where the relevant text available for abstraction in one publication [26]—“to continue to collect data until nothing new was being observed or recorded, no matter how long that takes”—was not accompanied by any term or label whatsoever.

This comparative analytic strategy (and our approach to analysis more broadly as described in strategy #7, below) is analogous to the process of *reciprocal translation*—a technique first introduced for meta-ethnography by Noblit and Hare [27] that has since been recognized as a common element in a variety of qualitative metasynthesis approaches [28]. Reciprocal translation, taken broadly, involves making sense of a study’s findings in terms of the findings of the other studies included in the review. In practice, it has been operationalized in different ways. Melendez-Torres and colleagues developed a typology from their review of the metasynthesis literature, describing four overlapping categories of specific operations undertaken in reciprocal translation: visual representation, key paper integration, data reduction and thematic extraction, and line-by-line coding [28]. The approaches suggested in both strategies #6 and #7, with their emphasis on constant comparison, appear to fall within the line-by-line coding category.

### Analysis

#### Generating credible and verifiable analytic interpretations

The analysis in a systematic methods overview must support its more general objective, which we suggested above is often to offer clarity and enhance collective understanding regarding a chosen methods topic. In our experience, this involves describing and interpreting the relevant literature in qualitative terms. Furthermore, any interpretative analysis required may entail reaching different levels of abstraction, depending on the more specific objectives of the review. For example, in the overview on sampling [18], we aimed to produce a comparative analysis of how multiple sampling-related topics were treated differently within and among different qualitative research traditions. To promote credibility of the review, however, not only should one seek a qualitative analytic approach that facilitates reaching varying levels of abstraction but that approach must also ensure that abstract interpretations are supported and justified by the source data and not solely the product of the analyst’s speculative thinking.

##### Principle #7:

Considering the qualitative nature of the analysis required in systematic methods overviews, it is important to select an analytic method whose interpretations can be verified as being consistent with the literature selected, regardless of the level of abstraction reached.

##### Strategy #7:

We suggest employing the constant comparative method of analysis [29] because it supports developing and verifying analytic links to the source data throughout progressively interpretive or abstract levels. In applying this approach, we advise a rigorous approach, documenting how supportive quotes or references to the original texts are carried forward in the successive steps of analysis to allow for easy verification.

**Fig. 2:**
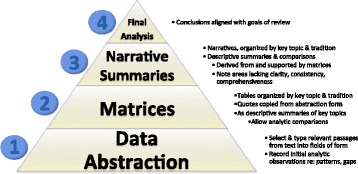
Summary of progressive steps of analysis used in the methods overview on sampling [18]

The analytic approach used in the methods overview on sampling [18] comprised four explicit steps, progressing in level of abstraction—data abstraction, matrices, narrative summaries, and final analytic conclusions (Fig. 2). While we have positioned *data abstraction* as the second stage of the generic review process (prior to Analysis), above, we also considered it as an initial step of analysis in the sampling overview for several reasons. First, it involved a process of constant comparisons and iterative decision-making about the fields to add or define during development and modification of the abstraction form, through which we established the range of concepts to be addressed in the review. At the same time, abstraction involved continuous analytic decisions about what textual quotes (ranging in size from short phrases to numerous paragraphs) to record in the fields thus created. This constant comparative process was analogous to open coding in which textual data from publications was compared to conceptual fields (equivalent to codes) or to other instances of data previously abstracted when constructing definitions to optimize their fit with the overall literature as described in strategy #6. Finally, in the data abstraction step, we also recorded our first interpretive thoughts in dedicated fields, providing initial material for the more abstract analytic steps.

In the second step of the analysis, we constructed topic-specific *matrices*, or tables, by copying relevant quotes from abstraction forms into the appropriate cells of matrices (for the complete set of analytic matrices developed in the sampling review, see Additional file 1 (matrices 3 to 10)). Each matrix ranged from one to five pages; row headings, nested three-deep, identified the methodological tradition, author, and publication, respectively; and column headings identified the concepts, which corresponded to abstraction fields. Matrices thus allowed us to make further comparisons across methodological traditions, and between authors within a tradition. In the third step of analysis, we recorded our comparative observations as *narrative summaries*, in which we used illustrative quotes more sparingly. In the final step, we developed analytic conclusions based on the narrative summaries about the sampling-related concepts within each methodological tradition for which clarity, consistency, or comprehensiveness of the available guidance appeared to be lacking. Higher levels of analysis thus built logically from the lower levels, enabling us to easily verify analytic conclusions by tracing the support for claims by comparing the original text of publications reviewed.

#### Integrative versus interpretive methods overviews

The analytic product of systematic methods overviews is comparable to qualitative evidence syntheses, since both involve describing and interpreting the relevant literature in qualitative terms. Most qualitative synthesis approaches strive to produce new conceptual understandings that vary in level of interpretation. Dixon-Woods and colleagues [30] elaborate on a useful distinction, originating from Noblit and Hare [27], between integrative and interpretive reviews. Integrative reviews focus on summarizing available primary data and involve using largely secure and well defined concepts to do so; definitions are used from an early stage to specify categories for abstraction (or coding) of data, which in turn supports their aggregation; they do not seek as their primary focus to develop or specify new concepts, although they may achieve some theoretical or interpretive functions. For interpretive reviews, meanwhile, the main focus is to develop new concepts and theories that integrate them, with the implication that the concepts developed become fully defined towards the end of the analysis. These two forms are not completely distinct, and “every integrative synthesis will include elements of interpretation, and every interpretive synthesis will include elements of aggregation of data” [30].

The example methods overview on sampling [18] could be classified as predominantly integrative because its primary goal was to aggregate influential authors’ ideas on sampling-related concepts; there were also, however, elements of interpretive synthesis since it aimed to develop new ideas about where clarity in guidance on certain sampling-related topics is lacking, and definitions for some concepts were flexible and not fixed until late in the review. We suggest that most systematic methods overviews will be classifiable as predominantly integrative (aggregative). Nevertheless, more highly interpretive methods overviews are also quite possible—for example, when the review objective is to provide a highly critical analysis for the purpose of generating new methodological guidance. In such cases, reviewers may need to sample more deeply (see strategy #4), specifically by selecting empirical research reports (i.e., to go beyond dominant or influential ideas in the methods literature) that are likely to feature innovations or instructive lessons in employing a given method.

## Conclusions

In this paper, we have outlined tentative guidance in the form of seven principles and strategies on how to conduct systematic methods overviews, a review type in which methods-relevant literature is systematically analyzed with the aim of offering clarity and enhancing collective understanding regarding a specific methods topic. Our proposals include strategies for delimiting the set of publications to consider, searching beyond standard bibliographic databases, searching without the availability of relevant metadata, selecting publications on purposeful conceptual grounds, defining concepts and other information to abstract iteratively, accounting for inconsistent terminology, and generating credible and verifiable analytic interpretations. We hope the suggestions proposed will be useful to others undertaking reviews on methods topics in future.

As far as we are aware, this is the first published source of concrete guidance for conducting this type of review. It is important to note that our primary objective was to initiate methodological discussion by stimulating reflection on what rigorous methods for this type of review should look like, leaving the development of more complete guidance to future work. While derived from the experience of reviewing a single qualitative methods topic, we believe the principles and strategies provided are generalizable to overviews of both qualitative and quantitative methods topics alike. However, it is expected that additional challenges and insights for conducting such reviews have yet to be defined. Thus, we propose that next steps for developing more definitive guidance should involve an attempt to collect and integrate other reviewers’ perspectives and experiences in conducting systematic methods overviews on a broad range of qualitative and quantitative methods topics. Formalized guidance and standards would improve the quality of future methods overviews, something we believe has important implications for advancing qualitative and quantitative methodology. When undertaken to a high standard, rigorous critical evaluations of the available methods guidance have significant potential to make implicit controversies explicit, and improve the clarity and precision of our understandings of problematic qualitative or quantitative methods issues.

A review process central to most types of rigorous reviews of empirical studies, which we did not explicitly address in a separate review step above, is *quality appraisal*. The reason we have not treated this as a separate step stems from the different objectives of the primary publications included in overviews of the methods literature (i.e., providing methodological guidance) compared to the primary publications included in the other established review types (i.e., reporting findings from single empirical studies). This is not to say that appraising quality of the methods literature is not an important concern for systematic methods overviews. Rather, appraisal is much more integral to (and difficult to separate from) the *analysis* step, in which we advocate appraising clarity, consistency, and comprehensiveness—the quality appraisal criteria that we suggest are appropriate for the methods literature. As a second important difference regarding appraisal, we currently advocate appraising the aforementioned aspects at the level of the literature in aggregate rather than at the level of individual publications. One reason for this is that methods guidance from individual publications generally builds on previous literature, and thus we feel that ahistorical judgments about comprehensiveness of single publications lack relevance and utility. Additionally, while different methods authors may express themselves less clearly than others, their guidance can nonetheless be highly influential and useful, and should therefore not be downgraded or ignored based on considerations of clarity—which raises questions about the alternative uses that quality appraisals of individual publications might have. Finally, legitimate variability in the perspectives that methods authors wish to emphasize, and the levels of generality at which they write about methods, makes critiquing individual publications based on the criterion of clarity a complex and potentially problematic endeavor that is beyond the scope of this paper to address. By appraising the current state of the literature at a holistic level, reviewers stand to identify important gaps in understanding that represent valuable opportunities for further methodological development.

To summarize, the principles and strategies provided here may be useful to those seeking to undertake their own systematic methods overview. Additional work is needed, however, to establish guidance that is comprehensive by comparing the experiences from conducting a variety of methods overviews on a range of methods topics. Efforts that further advance standards for systematic methods overviews have the potential to promote high-quality critical evaluations that produce conceptually clear and unified understandings of problematic methods topics, thereby accelerating the advance of research methodology.

## Additional file

Additional file 1: Submitted: Analysis_matrices. (DOC 330 kb)
