# Supplementary material for: Reviewing the research methods literature: principles and strategies illustrated by a systematic overview of sampling in qualitative research
Source: Syst Rev. 2016 Oct 11;5:172. doi: 10.1186/s13643-016-0343-0 (PMC5059917; doi:10.1186/s13643-016-0343-0)
Supplement: Additional file 1: — Submitted: Analysis_matrices. (DOC 330 kb) [file 13643_2016_343_MOESM1_ESM.doc]

# Matrices used in the overview of sampling in qualitative research [1]

### Table 1. Influential authors from three methodological traditions included in the analysis, their corresponding h-index scores and number of included publications.

| **Methodological tradition** | **Authors selected** | **H-index** | **Included publications** |
| --- | --- | --- | --- |
| Grounded theory | Anselm L. Strauss  Barney G. Glaser  Juliet M. Corbin  Kathy Charmaz  Adele Clarke | 70  44  34  28  21 | 3  3  1  2  1 |
| Phenomenology | Max van Manen (mixed)  Amadeo Giorgi (descriptive)  Paul Colaizzi (descriptive)  Marlene Cohen (hermeneutic)  Nancy Diekelman (hermeneutic) | 26  25  5  17  17 | 1  1  1  1  0 |
| Case study | Robert K. Yin  Robert E. Stake  Sharan B. Merriam | 35  32  31 | 2  3  1 |

### Table 2.a: Publications selected for inclusion in the analysis, listed by influential author and methodological tradition

| **Tradition** | **Author** | **Brief cit.** | **Full citation** |
| --- | --- | --- | --- |
| Grounded theory | Glaser | 1978 | Glaser, B. G. (1978). Theoretical sensitivity: Advances in the methodology of grounded theory. Mill Valley, CA: Sociology Press. |
|  |  | 1992 | Glaser, B. G. (1992). Basics of grounded theory analysis: Emergence versus forcing. Mill Valley, CA: Sociology Press. |
|  |  | 1998 | Glaser, B. G. (1998). Doing grounded theory: Issues and discussions. Mill Valley, CA: Sociology Press. |
|  | Strauss | 1967 | Glaser, B., & Strauss, A. (1967). The discovery of grounded theory. Chicago: Aldine. |
|  |  | 1973 | Schatzman, L., & Strauss, A. L. (1973). Field research: Strategies for a natural society (pp. 38-43). Englewood Cliffs, NJ: Prentice-Hall, Inc. |
|  |  | 1998 | Strauss, A.L., & Corbin, J. (1998). Basics of qualitative research: Techniques and procedures for developing grounded theory, 2nd ed. Thousand Oaks, CA: Sage Publications, Inc. |
|  | Corbin | 2008 | Corbin, J. & Strauss, A. (2008). Basics of qualitative research: Techniques and procedures for developing grounded theory (3rd ed.). Thousand Oaks, CA: Sage Publications, Inc. |
|  | Charmaz | 2003 | Charmaz, K. (2003). Grounded theory: Objectivist and constructivist methods. In N. K. Denzin & Y. S. Lincoln, eds. Strategies of Qualitative Inquiry, 2nd ed. [And In N. K. Denzin & Y. S. Lincoln, eds. (2000). The handbook of qualitative research. 2^nd^ ed.] Thousand Oaks, CA: Sage. |
|  |  | 2006 | Charmaz, K. (2006). Constructing grounded theory. Thousand Oaks, CA: Sage. |
|  | Clarke | 2005 | Clarke, A. E. (2005). Situational analysis: Grounded theory after the postmodern turn. Thousand Oaks, CA: Sage. |
| Phenomenology | van Manen | 1997 | van Manen, M. (1997). Researching lived experience: Human science for an action sensitive pedagogy (2nd edition). London, Ontario: Althouse Press. |
|  | Giorgi | 2009 | Giorgi, A. (2009). The descriptive phenomenological method in psychology; a modified Husserlian approach. Duquesne: Duquesne University Press. |
|  | Colaizzi | 1978 | Colaizzi, P. F. (1978). Psychological research as the phenomenologist views it. In: R. Vaile & M. King (Eds.), Existential phenomenological alternatives for psychology (pp. 48-71). New York: Oxford University Press. |
|  | Cohen | 2000 | Cohen, M. Z., Kahn, D.L., & Steeves, D.L. (2000). *Hermeneutic phenomenological research: A practical* *guide for nurse researchers*. Thousand Oaks, CA: Sage. |
| Case study | Yin | 2009 | Yin, R. K. (2009). Case study research: Design and methods (4th ed.). Thousand Oaks, CA: Sage. |
|  |  | 2011 | Yin, R. K. (2011). Qualitative research from start to finish. New York, NY: The Guilford Press. |
|  | Merriam | 2009 | Merriam, S. B. (2009). Qualitative research: A guide to design and implementation (revised and expanded from “Qualitative research and case study applications in education”). Hoboken, NJ: Jossey-Bass (Wiley). |
|  | Stake | 1995 | Stake, R. E. (1995). The art of case study research. Thousand Oaks, CA:  Sage. |
|  |  | 2005a | Stake, R. E. (2005a) Qualitative case studies. In Y. S. Lincoln & N. K. Denzin. Handbook of Qualitative Research (3rd ed., pp. 443-466). Thousand Oaks, CA: Sage. |
|  |  | 2005b | Stake, R. E. (2005b) Multiple case study analysis. New York, NY: The Guilford Press. |

### Table 2.b: Publications excluded from the analysis and reasons for exclusion, listed by influential author and methodological tradition

| **Tradition** | **Author** | **Citation** | **Reason excluded** |
| --- | --- | --- | --- |
| Grounded theory | Strauss | Strauss, A.L., & Corbin, J. (1990). Basics of qualitative research: Grounded theory procedures and techniques. Newbury Park, CA: Sage Publications, Inc. | Redundant, more recent used |
|  | Corbin | Corbin, J. Taking an analytic journey. In: Morse, J. M., Noerager Stern, P., Corbin, J., Bowers, B., Clarke, A. E., & Charmaz, K. C. (2009). Developing grounded theory: The second generation. Walnut Creek, CA: Left Coast Press. | Insufficient discussion of sampling |
|  |  | Corbin, J. & Strauss, A. (1990). Grounded theory research: procedures, canons, and evaluative criteria. *Qualitative Sociology*, 13, 3-21. | Insufficient discussion of sampling |
|  | Charmaz | Bryant, A. & Charmaz, K. (2007). Grounded theory research: Methods and practices, and: Grounded theory in historical perspective: An epistemological account. In Bryant, A. & Charmaz, K. (eds.). The Sage handbook of grounded theory. Thousand Oaks, CA: Sage. | Insufficient discussion of sampling |
|  | Clarke | Clarke, A. E. Chapter 5: Situational analysis. In: Morse, J. M., Noerager Stern, P., Corbin, J., Bowers, B., Clarke, A. E., & Charmaz, K. C. (2009). Developing grounded theory: The second generation. Walnut Creek, CA: Left Coast Press. | Insufficient discussion of sampling |
| Phenomenology | van Manen | van Manen, M. (1997). From meaning to method. Qualitative Health Research, 7(3), 345-369. | Insufficient discussion of sampling |
|  | Giorgi | Giorgi, A. (Ed.). (1985). Phenomenology and psychological research. Pittsburgh, PA: Duquesne University Press. | Redundant, more recent used |
|  |  | Giorgi, A. (1997). The theory, practice, and evaluation of the phenomenological method as a qualitative research procedure. J Phenomenological Psychology, 28(2), 235-260. | Redundant, more recent used |
|  |  | Giorgi, A. & Giorgi, B. (2003). The descriptive phenomenological psychological method. In P.M. Camic , J.E. Rhodes and L. Yardley (eds.) Qualitative research in psychology: Expanding perspectives in methodology & design (pp. 243-73). Washington, DC: American Psychological Association. | Redundant, more recent used |
|  | Diekelmann | Diekelmann N. & Ironside P. (1998). Hermeneutics. In Fitzpatrick J.J., ed. Encyclopedia of Nursing Research (pp. 243–245), New York, NY: Springer. | Insufficient discussion of sampling |
|  |  | Diekelmann N. (2001). Narrative pedagogy: Heideggerian hermeneutical analysis of lived experiences of students, teachers, and clinicians. *Adv Nursing Science*, 23, 53-71. | Primary research report |
|  | Cohen | Cohen, M. Z. & Omery, A. (1994). Schools of phenomenology: Implications for research. In J. M. Morse (Ed.). Critical Issues in Qualitative Research Methods (pp. 136-156). Thousand Oaks, CA: Sage Publications. | Insufficient discussion of sampling |
| Case study | Merriam | Merriam, S. (1988). Case study research in education: A qualitative approach. San Francisco: Jossey-Bass. | Redundant, more recent used |
|  |  | Merriam, S. (1998). Qualitative research and case study applications in education (revised and expanded from “Case study research in education: A qualitative approach”). San Francisco: Jossey-Bass. | Redundant, more recent used |
|  | Stake | Stake, R. E. (2000). Case studies. In N. K. Denzin & Y. S. Lincoln, eds. The handbook of qualitative research. 2^nd^ ed. [And In N. K. Denzin & Y. S. Lincoln, eds. (2003). Strategies of Qualitative Inquiry, 2nd ed.] Thousand Oaks, CA: Sage. | Redundant, more recent used |

### Table 3.a: Comparison of the use of the terms “sampling,” “sampling strategy,” and other terms used to stand for types of sampling across influential authors in three methodological traditions.

| **Tradition** | **Author** | **Ref.** | **“Sampling”** | | **Type of sampling** | | |
| --- | --- | --- | --- | --- | --- | --- | --- |
|  |  |  |  | | **1. “Sampling strategy”** | | **2. Other terms used to stand for types of sampling** |
|  |  |  | **Term used?** | **Definition** | **Term used?** | **Definition** | **Term and Definition (where applicable)** |
| Grounded theory | Glaser | 1978 | Yes | None | No | None | “*Sampling method*” |
|  |  | 1992 | Yes | None | No | None | None |
|  |  | 1998 | Yes | None | No | None | None |
|  | Strauss | 1967 | Yes | None | No | None | None |
|  |  | 1973 | Yes | “[The field researcher’s] *mapping tour (or tours) has provided him with sets of population or universes—of people, places, events, and any other categories determined to be of some importance. He cannot hope to observe everything, since “everything”—even for a large research team—is only a theoretical possibility, particularly for such complex phenomena as we are dealing with here. Hence selective sampling is a practical necessity*” (p. 38). This implies that sampling is a means to be selective about what to observe. | Yes | None | None |
|  |  | 1998 | Yes | The quote (p. 201), “*One of the major issues…is where to sample, that is where to go to obtain the data necessary…*” implies that sampling means obtaining the necessary data for one’s study. | No | None | None |
|  | Corbin | 2008 | Yes | None | No | None | None |
|  | Charmaz | 2003 | Yes | None | No | None | None |
|  |  | 2006 | Yes | None | Yes | “*Theoretical sampling is less of an explicit procedure than a strategy that you invoke and fit to your specific study*” (p. 107). | “*Sampling techniques*” (p. 100), eg: *“…sampling techniques for traditional quantitative research design.”* “*Forms of sampling*” is used interchangeably with “*sampling strategies*” in the same sentence (p. 102). |
|  | Clarke | 2005 | No | None | No | None | “*Data-gathering strategies*” (p. 75): “*Data sources must be specified explicitly, then enter the research design, and be collected… If the approach is too rigid, we will likely miss gathering important collectibles, and if too sloppy, we will likely end up unable to adequately defend some of our (likely favorite) arguments*” (p. 166-167). |
| Phenomenology | van Manen | 1997 | No | None | No | None | None |
|  | Giorgi | 2009 | No | None | No | None | None |
|  | Colaizzi | 1978 | No | None | No | None | None |
|  | Cohen | 2000 | Yes | “*Sampling implies that a researcher is choosing informants because those informants might have something to say about an experience they share with others*” (p. 45). | No | None | “*Method of choosing a sample*” (p. 45).  “*Approach*” (p. 51).  “*Frames*” from which to think about sampling (p. 46); three frames are discussed. |
| Case study | Yin | 2009 | Yes | “*The sampling logic requires an operational enumeration of the entire universe or pool of potential respondents and then a statistical procedure for selecting a specific subset of respondents to be surveyed*” p. (48). | No | None | None |
|  |  | 2011 | Yes | “[Another design choice] *involves the selection (or sampling) of the specific [data collection] units…to be included in a study. The task applies to the units at both the broader and narrower levels… Especially challenging are those studies that might have only a single data collection unit*” (p. 87). | No | None | “*Kinds of sampling*” (p. 88). Definitions for each “*kind of sampling*” provided in the glossary begin as follows: “*The selection of participants or sources of data to be used in a study, based on…*” This suggests that a “*kind of sampling*” is a rationale for selecting participants or data sources to be used in a study. |
|  | Merriam | 2009 | Yes | Applies to selecting cases and selecting data sources “*within the case.*” | Yes | None | “*Level of sampling*” (p. 81, 266-267), where one level is to “*select the case*,” and another is “*sampling within the case*.” Within a “*level of sampling*” Merriam describes using “*sampling strategies*” such as maximum variation sampling to select cases (p. 81). |
|  | Stake | 1995 | Yes | Applies to selecting cases and selecting data sources “*that best help us understand the case.*” | No | None | None |
|  |  | 2005a | Yes | Applies to selecting cases and selecting data sources. | No | None | None |
|  |  | 2005b | Yes | Applies to cases. | No | None | None |

### Table 3.b: Comparison of the use of the term “purposeful/purposive sampling” across influential authors in three methodological traditions

| **Tradition** | **Author** | **Ref.** | **Purposeful/purposive sampling** | |
| --- | --- | --- | --- | --- |
|  |  |  | **Term used** | **Definition** |
| Grounded theory | Glaser | 1978 | No | None |
|  |  | 1992 | No | None |
|  |  | 1998 | No | None |
|  | Strauss | 1967 | No | None |
|  |  | 1973 | No | None |
|  |  | 1998 | **Yes** | None |
|  | Corbin | 2008 | **Yes** | None |
|  | Charmaz | 2003 | No | None |
|  |  | 2006 | No | None |
|  | Clarke | 2005 | No | None |
| Phenomenology | van Manen | 1997 | No | None |
|  | Giorgi | 2009 | No | None |
|  | Colaizzi | 1978 | No | None |
|  | Cohen | 2000 | **Yes** | None |
| Case study | Yin | 2009 | No | None |
|  |  | 2011 | **Yes** | From Glossary (p. 311):  “*Purposive sample: The selection of participants or sources of data to be used in a study, based on their anticipated richness and relevance of information in relation to the study’s research questions. Richness and relevance include sources whose data are presumed to challenge and not just support a researcher’s thinking about the research questions and therefore should be part of the sample.*” |
|  | Merriam | 2009 | **Yes** | “*Purposeful sampling is based on the assumption that the investigator wants to discover, understand, and gain insight and therefore must select a sample from which the most can be learned.*” Merriam considers purposeful sampling synonymous with LeCompe & Preissle’s (1993)“*criterion-based selection*” (p. 77). |
|  | Stake | 1995 | No | None |
|  |  | 2005a | **Yes** | None |
|  |  | 2005b | **Yes** | “*For qualitative work, we will usually draw a purposive sample of cases, a sample tailored to our study; this will build in variety and create opportunities for intensive study*” (p. 24). |

### Table 4: Comparison of descriptions of saturation across influential authors in three methodological traditions

| **Tradition** | **Author** | **Ref.** | **Description of saturation** | **Term used?** | |
| --- | --- | --- | --- | --- | --- |
|  |  |  |  | **“Theoretical”** | **“Data”** |
| Grounded theory | Glaser | 1978 | Saturation is something one accomplishes through theoretical sampling, “*by looking for comparison groups*” (p. 37). And, “*Theoretical saturation of a category occurs when in coding and analyzing both no new properties emerge and the same properties continually emerge as one goes through the full extent of the data*” (p. 53). | Yes | No |
|  |  | 1992 | “*Theoretical sampling on any category ceases when it is saturated, elaborated and integrated in the emerging theory*” (p. 102). | No* | No |
|  |  | 1998 | No definition for saturation is given although Glaser uses the term: “*Thus theoretical sampling yields collection of data to the saturation of categories and their properties as it strikes out anew for theoretical completeness… Questions* [ie, interview questions] *constantly change with the requirements of the emergent theory and theoretical sampling. Once saturation occurs new questions must be asked pertinent to the new emergent issues of the main concern of the perhaps new interviewees…*” (p. 157-158).  “*Saturation brings dullness and boredom. …It is exciting until the whole substantive theory in the memos is saturated within the researcher’s sources of time and money and energy*” (p. 158). Although it is unclear, Glaser may mean that the theory can be expanded to more interesting conceptual areas as far as resources permit.  *“…data completeness is based only on theoretical completeness… There is no “N”, just sampling for saturation and completeness which yields a well integrated grounded substantive theory with parsimony and scope*” (p. 159). | No* | No |
|  | Strauss | 1967 | “*The criterion for judging when to stop sampling the different groups pertinent to a category is the category’s theoretical saturation. Saturation means that no additional data are being found whereby the sociologist can develop properties of the category. As he sees similar instances over and over again, the researcher becomes empirically confident that a category is saturated. He goes out of his way to look for groups that stretch diversity of data as far as possible, just to make certain that saturation is based on the widest possible range of data on the category*” (p. 61).  “*The adequate theoretical sample is judged on the basis of how widely and diversely the analyst chose his groups for saturating categories according to the type of theory he wished to develop*” (p. 63). “[But] *all categories are obviously not equally relevant, and so the depth of inquiry into each one should not be the same. Core theoretical categories, those with the most explanatory power, should be saturated as completely as possible… As his theory develops and becomes integrated, the sociologist learns which categories require the most and least complete saturation, and which ones can be dropped*” (p. 70). | Yes | No |
|  |  | 1973 | None | No | No |
|  |  | 1998 | Regarding theoretical saturation: “*This means until (a) no new or relevant data seem to emerge regarding a category, (b) the category is well developed in terms of its properties and dimensions demonstrating variation, and (c) the relationships among categories are well established and validated. …Unless a researcher gathers data until all categories are saturated, the theory will be unevenly developed and lacking density and precision*” (p. 212). | Yes | No |
|  | Corbin | 2008 | - Re data saturation: One “*continues to gather data until reaching the level of data ‘saturation.’ … Saturation is usually explained in terms of ‘when no new categories or relevant themes are emerging.’ But saturation is more than a matter of no new categories or themes emerging. It also denotes a development of categories in terms of their properties and dimensions, including variation, and possible relationships to other concepts*” (p. 148). - Re theoretical saturation: Although Corbin does not use the term theoretical saturation in the chapter on Theoretical Sampling, she describes something consistent with theoretical saturation: “*Eventually a researcher has to say this concept is sufficiently well developed for purposes of this research and accept what has not been covered as one of the limitations of the study*” (p. 149).  In a later chapter, Corbin defines theoretical saturation (p. 263): “*The point in analysis when all categories are well developed in terms of properties, dimensions, and variations. Further data gathering and analysis add little new to the conceptualization, though variations can always be discovered*.” | Yes | Yes |
|  | Charmaz | 2003 | “*Grounded theory researchers take the usual criteria of ‘saturation’ (i.e., new data fit into the categories already devised) of their categories for ending the research (Morse, 1995). But what does saturation mean? In practice, saturation seems elastic.*” Charmaz complains that published studies vary in this respect, and proposes that sustained field research rather than a handful of cases, “*more likely fulfills the criterion of saturation*” (p. 266-267). | No | No |
|  |  | 2006 | “*You conduct theoretical sampling by sampling to develop the properties of your category(ies) until no new properties emerge. Thus, you saturate your categories with data*” (p. 96). It is “*not the same as witnessing repetition of the same events or stories. … The common use of the term saturation refers to nothing new happening*” (p. 113).  Charmaz warns that early saturation may result from, “*Uncritical or limited analytic treatment.*” To support her “*concerns about foreclosing analytic possibilities and about constructing superficial analyses*,” she references Dey (1999) who has questioned the meaning and consequences of the concept of saturation (2006, p. 114-115). | No* | No |
|  | Clarke | 2005 | “*For a discourse study or discourse site within a multisite study, the data should offer both depth and range of variation. Using the grounded theory concept of saturation, narrative discourse data collection itself should continue until nothing analytically useful is being collected—until further analysis is no longer provoked by the new materials*” (p. 186). | No* | No |
| Phenomenology | van Manen | 1997 | None | No | No |
|  | Giorgi | 2009 | None | No | No |
|  | Colaizzi | 1978 | None | No | No |
|  | Cohen | 2000 | “*Ideally, a researcher would have the freedom and the resources to continue to collect data until nothing new was being observed or recorded, no matter how long that takes. But this is usually not the case, and the hermeneutic phenomenological researcher must rely on previous studies and clinical experience*” (p. 55, para 4). Although the word saturation is not mentioned, this implies that data saturation usually cannot be achieved in a phenomenology study. | No | No** |
| Case study | Yin | 2009 | None | No | No |
|  |  | 2011 | None | No | No |
|  | Merriam | 2009 | “*Lincoln and Guba (1985) recommend sampling until a point of saturation or redundancy is reached*” (p. 80). This suggests data saturation. | No | No** |
|  | Stake | 1995 | None | No | No |
|  |  | 2005a | None | No | No |
|  |  | 2005b | None | No | No |

*The description of saturation is consistent with what has been described elsewhere as theoretical saturation although the term was not used.

**The term is not used, but data saturation seems to be implied.

Full unedited matrix (all traditions):

| **Tradition** | **Author** | **Reference** | **Description of saturation** | **Descriptors used?** | |
| --- | --- | --- | --- | --- | --- |
|  |  |  |  | **“Theoretical”** | **“Data”** |
| Grounded theory | Glaser | 1978 | “Only as he discovers codes and tries to saturate them by looking for comparison groups, does both what codes and their properties and where to collect data on them emerge” (p. 37 para 2).  “Theoretical saturation of a category occurs when in coding and analyzing both no new properties emerge and the same properties continually emerge as one goes through the full extent of the data” (p. 53, para 2). | Yes | No |
|  |  | 1992 | “Theoretical sampling on any category ceases when it is saturated, elaborated and integrated in the emerging theory” (p. 102, para 1). | No | No |
|  |  | 1998 | “Thus theoretical sampling yields collection of data to the saturation of categories and their properties as it strikes out anew for theoretical completeness… Questions [ie, interview questions] constantly change with the requirements of the emergent theory and theoretical sampling. Once saturation occurs new questions must be asked pertinent to the new emergent issues of the main concern of the perhaps new interviewees.”  [Note, Glaser’s definition of saturation here is not stated.]  “Saturation brings dullness and boredom. Theoretical sampling keeps up the motivation to continue on with data collections…It is exciting until the whole substantive theory in the memos is saturated within the researcher’s sources of time and money and energy” (p. 158, para 3).  [How can a theory be saturated within resources???]  “…data completeness is based only on theoretical completeness… There is no “N”, just sampling for saturation and completeness which yields a well integrated grounded substantive theory with parsimony and scope” (p. 159, para 2).  [INTERPRETIVE NOTE: From this, Cathy and I agreed that the interpretation was (2011-01-13): Although categories may become saturated, theoretical sampling keeps pointing to new directions to sample, expanding the scope of the theory (and study); this can continue as long as one wants, until the researcher has used up all resources (time, money, energy).] | No* | No |
|  | Strauss | 1967 | “The criterion for judging when to stop sampling the different groups pertinent to a category is the category’s *theoretical saturation*. *Saturation* means that no additional data are being found whereby the sociologist can develop properties of the category. As he sees similar instances over and over again, the researcher becomes empirically confident that a category is saturated. He goes out of his way to look for groups that stretch diversity of data as far as possible, just to make certain that saturation is based on the widest possible range of data on the category” (p. 61).  “The adequate theoretical sample is judged on the basis of how widely and diversely the analyst chose his groups for saturating categories according to the type of theory he wished to develop” (p. 63, para 2). | Yes | No |
|  |  | 1973 | - | No | No |
|  |  | 1998 | Regarding theoretical saturation: “This means until (a) no new or relevant data seem to emerge regarding a category, (b) the category is well developed in terms of its properties and dimensions demonstrating variation, and (c) the relationships among categories are well established and validated. …Unless a researcher gathers data until all categories are saturated, the theory will be unevenly developed and lacking density and precision” (p. 212). | Yes | No |
|  | Corbin | 2008 | One “continues to gather data until reaching the level of data ‘saturation.’ … Saturation is usually explained in terms of ‘when no new categories or relevant themes are emerging.’ But saturation is more than a matter of no new categories or themes emerging. It also denotes a development of categories in terms of their properties and dimensions, including variation, and possible relationships to other concepts” (p. 148, 2^nd^ last para). “Eventually a researcher has to say this concept is sufficiently wee developed for purposes of this research and accept what has not bee covered as one of the limitations of the study” (p. 149, para 1).  Theoretical saturation:  She does not use the term (in Ch. 7), but describes what Glaser has called theoretical sampling (above). Later, in Chapter 12 about Integrating Categories, she defines this term (p. 263): “The point in analysis when all categories are well developed in terms of properties, dimensions, and variations. Further data gathering and analysis add little new to the conceptualization, though variations can always be discovered.” [**NOTE: because of Corbin’s inconsistent? description, the difference between data and theoretical saturation is not made obvious to the reader.**] | Yes | Yes |
|  | Charmaz | 2003 | “Grounded theory researchers take the usual criteria of ‘saturation’ (i.e., new data fit into the categories already devised) of their categories for ending the research (Morse, 1995). But what does saturation mean? In practice, saturation seems elastic” (p. 266-267). Charmaz goes on to warn against how studies may still be arbitrarily ended early, and proposes that a truly saturated study is more likely based on a “sustained field of research… [that] has the resonance of intimate familiarity with the studied world.” | No | No |
|  |  | 2006 | “You conduct theoretical sampling by sampling to develop the properties of your category(ies) until no new properties emerge. Thus, you *saturate* your categories with data” (p. 96).  It is “not the same as witnessing repetition of the same events or stories. … The common use of the term saturation refers to nothing new happening. ‘I kept finding the same patterns’” (p. 113).  On page 114, Charmaz questions how meaningful the concept of theoretical saturation is, and does not offer positive recommendations per se. | Yes | No |
|  | Clarke | 2005 | For a discourse study or discourse site within a multisite study, the data should offer both depth and range of variation. Using the grounded theory concept of saturation, narrative discourse data collection itself should continue until *nothing analytically useful* is being collected—until further analysis is no longer provoked by the new materials” (p. 186, para 1). | No* | No |
| Phenomenology | van Manen | 1997 | - | No | No |
|  | Giorgi | 2009 | - | No | No |
|  | Colaizzi | 1978 | - | No | No |
|  | Cohen | 2000 | “Ideally, a researcher would have the freedom and the resources to continue to collect data until nothing new was being observed or recorded, no matter how long that takes. But this is usually not the case, and the hermeneutic phenomenological researcher must rely on previous studies and clinical experience” (p. 55, para 4). [The word saturation is not mentioned, however. This implies that saturation is usually not achieved by the data in phenomenology.] | No | No** |
| Case study | Yin | 2009 | - | No | No |
|  |  | 2011 | - | No | No |
|  | Merriam | 2009 | “Lincoln and Guba (1985) recommend sampling until a point of saturation or redundancy is reached” (p. 80). This suggests data saturation. | No | No** |
|  | Stake | 1995 | - | No | No |
|  |  | 2005a | - | No | No |
|  |  | 2005b | - | No | No |

*The description of saturation matches what has been described for theoretical saturation although the term was not used.

**The term is not used, but data saturation seems to be implied.

### Table 5: Comparison of recommendations regarding sample size across influential authors in three methodological traditions

| **Tradition** | **Author** | **Ref.** | **Recommendations** | **Numbers suggested** |
| --- | --- | --- | --- | --- |
| Grounded theory | Glaser | 1978 | None | None |
|  |  | 1992 | None | None |
|  |  | 1998 | Theoretical sampling does not result in a representative sample. “*But if a researcher submits a proposal to be funded by a board which requires proposals based on numbers, populations and deadlines, then it is a small price to pay to recast the research proposal in these terms so it can be understood and hopefully funded*” (p. 159). | None |
|  | Strauss | 1967 | “*In research carried out for discovering theory, the sociologist cannot cite the number and types of groups from which he collected data until the research is completed*” (p. 50). | None |
|  |  | 1973 | None | None |
|  |  | 1998 | None | None |
|  | Corbin | 2008 | “*It is doubtful that five or six one-hour interviews can lead to saturation*” (p. 149). | >6 interviews |
|  | Charmaz | 2003 | None | None |
|  |  | 2006 | “*A small study with modest claims might allow proclaiming saturation early. …A study of 25 interviews may suffice for certain small projects but invites skepticism when the author’s claims are about, say, human nature or contradict established research*” (p. 114). | ≥25 interviews |
|  | Clarke | 2005 | None | None |
| Phenomenology | van Manen | 1997 | None | None |
|  | Giorgi | 2009 | None | None |
|  | Colaizzi | 1978 | “*Regarding the number of subjects selected, this depends on various factors that must be tried out in each research project. In this research I used 12 subjects*” (p. 58). | Example:  12 participants |
|  | Cohen | 2000 | Estimates were suggested by examples (Cohen, 2000, p. 56): *“Interviewing at least five would not ensure that I would have a survivor in my sample, but it would increase my chances. I wanted to follow fewer than 10 because I wanted to follow each informant intensely rather than spread my attention over a larger group…A review of the literature on caregivers of dying patients led me to believe that the experience was not as intense and variable day to day as having a bone marrow transplant. Thus, I believed I could use a larger sample, 30 at least.”*  Providing information about sample size, “*is not at odds with the philosophical underpinnings of the hermeneutic phenomenological method*.” If an experience is more intense or variable, then a smaller sample, which is followed more intensely, is suggested. “*The scientifically important criterion for determining sample size for the hermeneutic phenomenological researcher is the intensity of the contact needed to gather sufficient data regarding a phenomenon or experience. This intensity is measured in both length of time it takes for an event to occur, …and how often a participant should be contacted to understand the changes undergone*” (p. 56). | Examples:  <10 participants (followed intensely),  30 participants (followed less intensely) |
| Case study | Yin | 2009 | None | None |
|  |  | 2011 | “*Larger numbers can be better than smaller numbers because a larger number can create greater confidence in a study’s findings*” (p. 89). Regarding the “*narrower level*” at which sampling can occur: “*The number of interviewees, practices, policies, or actions included in a study can easily fall in the range of 25-50 such units*…” This depends on, “*the complexity of your study topic and the depth of data collection from each unit*” (p. 91). | 25-50 units  (interviewees, practices, policies, or actions) |
|  | Merriam | 2009 | Regarding the number of cases in a multiple case study: “*The more cases included in a study, and the greater the variation across the cases, the more compelling an interpretation is likely to be*” (p. 49). And, “*The inclusion of multiple cases is, in fact, a common strategy for enhancing the external validity or generalizability of your findings*” (p. 50). Whereas, “*The size of the sample within the case is determined by a number of factors relevant to the study’s purpose*” (p. 82). |  |
|  | Stake | 1995 | None | None |
|  |  | 2005a | None | None |
|  |  | 2005b | “*The benefits of multicase study will be limited if fewer than, say, 4 cases are chosen, or more than 10. … 15 or 30 cases provide more uniqueness of interactivity than the research team and readers can come to understand. But for good reason, many multicase studies have fewer than 4 or more than 15 cases*” (p. 22). | 4-10 cases |

### Table 6: Comparison of definitions of theoretical sampling and descriptions of what can be selected across influential authors in grounded theory

| **Tradition** | **Author** | **Ref.** | **Definition** | **What can be selected (sampling units)** | **Comments on using secondary data** | **Comments on sampling to move from substantive to formal theory** |
| --- | --- | --- | --- | --- | --- | --- |
| Grounded theory | Glaser | 1978 | The original Glaser & Strauss (1967) definition is quoted (see below). Also, “*Theoretical sampling on any category ceases when it is saturated, elaborated and integrated in the emerging theory*” (p. 36). | “*Groups are chosen as they are needed rather than before the research begins. Theoretical purpose, as a criterion, is embodied in generated ideas or ideas deduced from them. These ideas are properties of groups not the group itself or its most apparent description. Thus apples can be compared to oranges if the comparison is the kinds of vitamins found beneath and in the skin. The premature baby ward can be compared with a cancer ward on the criterion of awareness*” (p. 42). Glaser is not more concrete about what “*groups*” can be.  And in Chapter 1 (p. 8): “*The hard thought necessary to generate good ideas requires that the analyst treat “all as data” at some level. Whether his material is research data, others ideas on it or the literature, it is to be compared to the ongoing data and memos for the purpose of generating the best fitting and working idea.*” | “*The grounded theorist simply theoretically samples the data that has been obtained, by ‘appreciating what he has, not what the project did not collect’*” (p. 53-54). | “*An important rule is, when the analyst is still ‘young’ at generating skills, as a sociologist and/or as a scholar within the area, to sample exclusively within the substantive area until focus on a basic social psychological problem and the process by which it is resolved both have been discovered and stabilized in an emerging theoretical framework. Once this has occurred then it is safe from undermining of relevance of core process to sample outside the substantive area of study to further elaborate the emerging theory*” (p. 50). |
|  |  | 1992 | The 1978 definition above is repeated (p. 101-102). | Not described | None | None |
|  |  | 1998 | None (assumes prior knowledge) | “*Theoretical sampling takes all as data… Different slices of data can abound: interviews, casual comments, observations, reports, manuals, files newspaper articles, tables, charts etc, etc… Although interviews are typical* […limiting a study to collecting only this type of data] *is constraining of theoretical sampling and theory generation*.” (p. 159). | None | None |
|  | Strauss | 1967 | “*Theoretical sampling is the process of data collection for generating theory whereby the analyst jointly collects, codes, and analyzes his data and decides what data to collect next and where to find them, in order to develop his theory as it emerges. This process of data collection is controlled by the emerging theory, whether substantive or formal*” (p. 45). “*The researcher chooses any groups that will help generate, to the fullest extent, as many properties of the categories as possible, and will help relate categories to each other and to their properties*” (p. 49). | Sampling of “*groups*” is most commonly mentioned. For example: “*In short, how does the sociologist select multiple comparison groups?*” Groups are defined in a footnote as “*aggregates or single people*” (p. 47). Sampling at higher levels is possible: “*Rough estimates can be given of how many large units (such as number of cities, regions, and countries) will be sampled*” (p. 74). Choosing different data types is described, for example: “*historical documents, or other library materials*” (p. 53). And, “*no one kind of data on a category nor technique for data collection is necessarily appropriate. Different kinds of data give the analyst different views or vantage point from which to understand a category and to develop its properties; these different views we have called slices of data*” (p. 65). | “*Theoretical sampling can be done with previously collected research data*… [This] *amounts to collecting data from collected data*” (p. 71). | “*The simplest comparisons are, of course, made among different groups of exactly the same substantive type; for instance, federal book-keeping departments. These comparisons lead to a substantive theory that is applicable to this one type of group. … The scope of a substantive theory can be carefully increased and controlled by such conscious choices of groups*” (p. 52). Later, the authors illustrate how this can be done to satisfy the researcher’s desire “*to begin generating a formal theory*” or a “*mixture of both*” (ie, formal and substantive theory; p. 54). An example is given from Glaser and Strauss’ *Awareness of Dying* study in which the scope was broadened by sampling in Malay and other Asian hospitals (p. 57-60). |
|  |  | 1973 | Not described | NA | NA | NA |
|  |  | 1998 | “*Data gathering driven by concepts derived from the evolving theory and based on the concept of “making comparisons,” whose purpose is to go to places, people, or events that will maximize opportunities to discover variations among concepts and to densify categories in terms of their properties and dimensions*” (p. 201). | “*Sample incidents, events, or happenings and not persons per se. Incidents or events represent situations…that are somehow problematic or routine and to which there is a response through some form of action/interaction*” (p. 202). As well, one can sample archival material such as “*data from a library*” (p. 212). | “*It is customary to call the analysis of* [the collected interviews or field notes of another researcher] *by the term ‘secondary data analysis.’ A researcher building theory can code these materials as well, employing theoretical sampling in conjunction with the usual coding procedures*” (p. 213). | None |
|  | Corbin | 2008 | “A *method of data collection based on concepts-themes derived from data. The purpose of theoretical sampling is to collect data from places, people, and events that will maximize opportunities to develop concepts in terms of their properties and dimensions, uncover variations, and identify relationships between concepts*” (p. 143). | “*It is concepts that are sampled in data. Participants provide the data that tell us about those concepts. So, when researchers sample theoretically they go to places, persons, and situations that will provide information about the concepts*” (p. 144). Data types sampled can also include “*historical data*” (p. 147) or “*documents, memoirs, biographies, audio- or videotapes*” (p. 151). | On p. 150: “*Using already collected data can be done… Questions about a concept(s) serve as a guide for what incidents to look for in the next set of data.*” And “*It is not unusual to return to previously analyzed data and look at them with a fresh eye*.” | “*Analyzing data for process at a formal theory level*,” is discussed on p. 102: “*One begins with a concept such as “awareness” or “stigma” and samples theoretically, but in this case compares and contrasts data across research contexts*.” |
|  | Charmaz | 2003 | “*Then we go back to the field and collect delimited data to fill those conceptual gaps and holes—we conduct theoretical sampling. At this point we choose to sample specific issues only; we look for precise information to shed light on the emerging theory. …Theoretical sampling…relies on the comparative methods within grounded theory…to develop our emerging categories and to make them more definitive and useful. Thus the aim of sampling is to refine ideas*” (p. 265). | “*As we gather rich data, we draw from multiple sources—observations, conversations, formal interviews, auto-biographies, public records, organizational reports, respondents’ diaries and journals, and our own tape-recorded reflections*” (p. 258). And “*Although we often sample people, we may sample scenes, events, or documents, depending on the study and where the theory leads us. We may return to the same settings or individuals to gain further information*” (p. 265). | None | “*Theoretical sampling is a pivotal part of the development of formal theory. …Thus we could seek comparative data in substantive areas through theoretical sampling to help us tease out less visible properties of our concepts and the conditions and limits of their applicability*” (p. 266). |
|  |  | 2006 | Glossary definition (p. 189): “*A type of grounded theory sampling in which the researcher aims to develop the properties of his or her developing categories or theory, not to sample randomly selected populations or to sample representative distributions of a particular population. When engaging in theoretical sampling, the researcher seeks people, events, or information to illuminate and define the boundaries and relevance of the categories. Because the purpose of theoretical sampling is to sample to develop the theoretical categories, conducting it can take the researcher across substantive areas.*” Goals for theoretical sampling are to: “*delineate the properties of a category; check hunches about categories; saturate the properties of a category; distinguish between categories; clarify relationships between emerging categories; identify variation in a process*” (p. 104, bullets). | From Glossary definition for theoretical sampling (p. 189): “*When engaging in theoretical sampling, the researcher seeks people, events, or information to illuminate and define the boundaries and relevance of the categories*.” | None | “*The logic of grounded theory can reach across substantive areas and into the realm of formal theory*“ (p. 8). “*Because the purpose of theoretical sampling is to sample to develop the theoretical categories, conducting it can take the researcher across substantive areas*” (p. 189). |
|  | Clarke | 2005 | None | “*Data that address these questions* [ie, social worlds/arenas maps] *can be generated in heterogeneous ways: interviews, organizational documents, historical as well as contemporary archives, observations at meetings or other gatherings of key actors, secondary data (previous historical and contemporary research on the topic, media imagery and discourses), and so on*” (p. 113). | “*Data that address these questions can be generated in heterogeneous ways: interviews, organizational documents, historical as well as contemporary archives, observations at meetings or other gatherings of key actors, secondary data (previous historical and contemporary research on the topic, media imagery and discourses), and so on*” (p. 113). | None |

NA: not applicable.

### Table 7: Comparison of recommendations regarding a priori or initial sampling across influential authors in grounded theory

| **Tradition** | **Author** | **Ref.** | **A priori or initial sampling** | | |
| --- | --- | --- | --- | --- | --- |
|  |  |  | **Described** | **Acceptable for GT?** | **Term used and definition** |
| Grounded theory | Glaser | 1978 | Yes | Unclear | “Selective sampling” (Schatzman & Strauss, 1973, is referenced): “*Selective sampling refers to the calculated decision to sample a specific locale according to a preconceived but ‘reasonable’ initial set of dimensions, (such as time, space, identity or power) which are worked out in advance for a study*” (p. 37).  “Initial strategies”: “*Several analysts start their field work with initial strategies. … Based on ‘local’ concepts, they will go to the groups which they believe will maximize the possibilities of obtaining data and leads for more data on their question. Thus it was natural to start the dying study on an intensive care unit and a cancer ward. They will also begin by talking with the most knowledgeable people to get a line on relevancies and leads to track down more data and where and how to locate oneself for a rich supply of data*” (p. 45). |
|  |  | 1992 | Yes | No | “Conventional sampling”: “*In conventional sampling the analyst questions, guesses and uses experience to go where he thinks he will have the data to test his hypotheses and find the theory that he has preconceived*” (p. 105). |
|  |  | 1998 | No | NA | None |
|  | Strauss | 1967 | Yes | Yes | “Decisions concerning initial collection of data”: “*Beyond the decisions concerning initial collection of data, further collection cannot be planned in advance of the emerging theory*” (p. 47; see also p. 59 for an example of how making decisions about initial data collection was acceptable in Glaser and Strauss’ own grounded theory research). |
|  |  | 1973 | Yes | Unclear | “Selective sampling”: “*to move from one selected sub-site to another and sample at each site various “dimensions”: time, space, people and events*” (p. 39). Suggesting that this is not only an initial form of sampling, the authors state: “*other categories (such as age, sex, status, role or function in the organization, or even stated philosophy or ideology) might operate as a departures for selective sampling. Our researcher is quite aware that his own activity might, and probably will, lead to discoveries of new categories. Hence those regarded earlier of relative unimportance may become extremely important…consequently he develops appropriate hypotheses and so plans more thorough sampling by that particular dimension*” (p. 42). |
|  |  | 1998 | Yes | Yes | “Initial sampling: “*In the initial sampling, the researcher is interested in generating as many categories as possible; hence, he or she gathers data in a wide range of pertinent areas*” (p. 203). |
|  | Corbin | 2008 | Yes | Yes | [no term used] “*At the beginning of a study there are many sampling matters that the research must consider. … Decisions made about the site or group to study…directed by the main research question*” (p. 151). |
|  | Charmaz | 2003 | Yes | Yes | None |
|  |  | 2006 | No | NA | “Initial sampling”: “*For initial sampling, you establish sampling criteria for people, cases, situations, and/or settings before you enter the field*” (p. 100). |
|  | Clarke | 2005 | Yes | Yes | [no term used] “*While also relying on traditional theoretical sampling, I am arguing that we also need to design our research from the outset in order to explicitly gather data about theoretically and substantively underdeveloped areas that may lie in our situations of inquiry*” (p. 76). Sensitizing concepts frame “*directions along which to look but not what to see*…[nor] *what not to see*” (p. 77). |

GT: grounded theory. NA: not applicable.

### Table 8: Comparison of sampling units and types of sampling described by influential authors in phenomenology and case study

| **Tradition** | **Author** | **Ref.** | **Sampling units  (data collection units)** | **Type(s) of sampling discussed and brief description(s)** |
| --- | --- | --- | --- | --- |
| Phenomenology | van Manen | 1997 | People only | Generic comment (no types of sampling): “*For example, depending on the nature of the project and the stage of the inquiry process, the conversational interview method may serve either to mainly gather lived-experience material (stories, anecdotes, recollections of experiences, etc.) or serve as an occasion to reflect with the partner (interviewee) of the conversational relation on the topic at hand*” (p. 63). |
|  | Giorgi | 2009 | People; descriptions of situations (manifesting the experience) within the same person | 1. “Obtaining concrete descriptions of experiences from others”: “*The descriptions are given by ordinary persons within the natural attitude. The situations to be described are selected by the participants themselves and what is sought is simply a description that is as faithful as possible to the actual lived-through event. This description is referred to as the raw data, and all of it has to be accounted for*” (p. 66). “…*the question the researcher poses should invite the interviewee to focus upon a specific situation that he or she actually experienced*” (p. 124). 2. Selecting participants based on the researcher-participant relationship: “*If a researcher wants a deeply self-revealing experience to be articulated, then perhaps an intimate friend ought to be the participant (obviously, with anonymity guaranteed), or else the researcher ought to establish a relationship greater than that of passing acquaintanceship. If one is seeking a rather ordinary experience (for example, a learning situation), then strangers could be participants, but still, a certain degree of rapport should be established*” (p. 123). |
|  | Colaizzi | 1978 | People only | “Selecting subjects”: “*Anyone can serve as subjects provided they have experienced the [phenomenon of interest] and are able to intelligently communicate it… Experience with the investigated topic and articulateness suffice as criteria for selecting subjects*” (p. 58). |
|  | Cohen | 2000 | People; descriptions of experiences within the same person | 1. Sampling “experiences of place”: “*For the hermeneutic phenomenological researcher, it is useful to look at places and how the experience of place is a part of the phenomenon in which we are interested*” (p. 47). “*But how does a researcher sample to learn about a place? The obvious answer is by being there*.” Cohen goes on to describe how his team observed and took field notes in one study, but provides no indication of how decisions were made about what to observe (p. 47-48). 2. Selecting informants based on characteristics: “*One of the tenets of hermeneutic phenomenological research is to see informants not in terms of groups of individual characteristics that can be seen as variables but as people who offer a picture of what it is like to be themselves as they make sense of an important experience… care should be taken that picking out characteristics...to guide sampling does not oversimplify the complex human world that affects the way people interpret their experiences*.” More desirable characteristics are, “*better with language*,” “*introspective*,” and “*willing to tell*” (p. 50). 3. “Two-tier system” for selecting informants: The first tier of participants is selected via referrals to people who underwent the experience in a unique way (thus capturing variety), and then interviewed according to a pre-planned protocol schedule; a smaller second tier of participants is selected from first tier based on the criterion that their preliminary interviews contain important themes—these participants are followed more intensively. This approach allows one to capture depth on some key themes, but also to capture variety because of the larger sample of participants followed less intensively (p. 51). 4. Selecting informants who share a language “tradition”: “*Those people who share a tradition or a way of talking about experiences are people who share a specialized knowledge about a set of rules and options that govern how they can talk in specific social situations*” (p. 52). “*Exploring who shares a way of talking about a particular experience cannot be determined before the researcher enters the field*” (p. 54). “*Determining who shares a way of talking about experiences is problematic and can be determined only through careful data collection*” (p. 55). |
| Case study | Yin | 2009 | Cases | “Screening the candidate cases”: “*When you have only a score or so (20 to 30) of possible candidates that can serve as your cases…, the screening may consist of querying people knowledgeable about each candidate. You may even collect limited documentation about each candidate… If doing a single-case study, choose the case that is likely, all other things being equal, to yield the best data*” (p. 91). For Yin, single cases may represent the critical case, an extreme or unique case, the representative or typical case, the revelatory case, or longitudinal case (p. 47-49).   - “Replication designs”: “*If doing a multiple-case study, select cases that best fit your (literal or theoretical) replication design*” (p. 91). In replication designs, “*Each case must be carefully selected so that it either (a) predicts similar results (a literal replication) or (b) predicts contrasting results but for predictable reasons (a theoretical replication)*” (p. 54-55). - Two-stage screening procedure: With more than 20-30 candidate cases, the above screening procedure is preceded by an earlier stage that, “*should consist of collecting relevant quantitative data about the entire pool, from some archival source. … Once obtained, you should define some relevant criteria for either stratifying or reducing the number of candidates* [to 20-30]” (p. 92). |
|  |  | 2011 | Not directed specifically at case study: “*Units at the broader level are usually some kind of geographic, organizational, or social entity. The units at the narrower level frequently consist of participants. However, the narrower level also can have policies, practices, or actions as units*” (p. 82). | Not directed specifically at case study:   1. Purposive sampling: “*The selection of participants or sources of data to be used in a study, based on their anticipated richness and relevance of information in relation to the study’s research questions. Richness and relevance include sources whose data are presumed to challenge and not just support a researcher’s thinking about the research questions and therefore should be part of the sample*” (p. 311). “*Purposive sampling differs from several other kinds of sampling, snowball sampling, and random sampling*” (p. 88). 2. Convenience sampling: “*selecting data collection units simply because of their ready availability—normally is not preferred. It is likely to produce an unknown degree of incompleteness because the most readily available sources of data are not likely to be the most informative sources. Similarly, convenience samples are likely to produce an unwanted degree of bias*” (p. 88). 3. Snowball sampling: “*selecting new data collection units as an offshoot of existing ones—can be acceptable if the snowballing is purposeful, not done out of convenience. In the course of an interview you might learn of other persons who can be interviewed. The snowballing occurs when you follow such a lead and let those new ones result in identifying yet other possible interviewees*” (p. 89). 4. Collecting objects: “*Objects are likely to exist in great abundance… collecting documents and records…can be time-consuming… Two tactics can help… First, get an idea of the full array of any type of object to be collected… Also get an idea of the difficulty you will have in accessing and retrieving these objects… Second, after doing some preliminary collecting, immediately review the resulting data… You may then decide to invest less (or even more) time in the collection effort*” (p. 148-9). |
|  | Merriam | 2009 | Cases;  Units within the case: sites, events, activities, people, documents | 1. Selecting the case: “*you would first establish the criteria that will guide case selection and then select a case that meets those criteria. …For multicase or comparative case studies you would select several ‘cases’ based on relevant criteria. One of the criteria might be that you want as much variation as possible; hence, you would be employing a maximum variation sampling strategy*” (p. 81) For research proposals: “*If you are doing a case study you will have two levels of purposeful sampling. First, tell us the criteria for selecting the case(s)*” (p. 267; the second level is sampling within the case). 2. Sampling within the case: “*A sample within the case needs to be selected either before the data collection begins or while the data are being gathered (ongoing or theoretical sampling). Random sampling can be used within the case, and indeed, this is one strategy that can be employed for addressing validity… However, a second set of criteria is usually needed to purposefully select whom to interview, what to observe, and which documents to analyze*” (p. 81-2). 3. Purposive/purposeful/criterion-based sampling: “*purposeful sampling… is used to select the sample within the case, just as it is used to select the case itself*” (p. 82). And, “*the most appropriate sampling strategy is non-probabilistic—the most common form of which is called purposive (Chein, 1981) or purposeful (Patton, 2002). … LeCompte and Preissle (1993, p. 69) prefer the term criterion-based selection to the terms purposive or purposeful sampling… To begin purposive sampling, you must first determine what selection criteria are essential in choosing the people or sites to be studied*” (p. 77). “*Types of purposeful sampling*” include (p. 77-80):  - Typical: “*selected because it reflects the average person, situation, or instance of the phenomenon of interest*.” - Unique: “*based on unique, atypical, perhaps rare attributes or occurrences of the phenomenon of interest*.” - Maximum variation: “*first identified by Glaser and Strauss (1967)… A grounded theory, it was reasoned, would be more conceptually dense and potentially more useful if it had been ‘grounded’ in widely varying instances of the phenomenon. …Sometimes this strategy involves ‘a deliberate hunt for negative’ or disconfirming ‘instances or variations’ of the phenomenon* (Miles and Huberman, 1994, p. 29).” - Snowball/chain/network: “*perhaps the most common form of purposeful sampling. This strategy involves locating a few key participants who easily meet the criteria you have established for participation in the study. As you interview these early participants you ask each one to refer you to other participants*.” - Ongoing/Theoretical: “*The researcher begins with an initial sample chosen for its obvious relevance to the research problem. The data lead the investigator to the next document to be read, the next person to be interviewed, and so on. It is an evolving process guided by the emerging theory. …As data are being collected and theoretical constructs begin to evolve, the researcher might also look for exceptions (negative-case selection) or variants (discrepant-case selection) to emerging findings*.”  1. Probability (random) sampling: “*Probability sampling (of which simple random sampling is the most familiar example) allows the investigator to generalize results of the study from the sample to the population from which it was drawn. Since generalization in a statistical sense is not a goal of qualitative research, probabilistic sampling is not necessary or even justifiable in qualitative research*” (p. 77). And yet Merriam states, “*Random sampling can be used within the case, and indeed, this is one strategy that can be employed for addressing validity. Most commonly, however, purposeful sampling as outlined earlier is used to select the sample within the case*” (p. 82). |
|  | Stake | 1995 | Cases;  Units within the case: people, places, occasions | 1. Selecting cases: “*It may be useful to try to select cases which are typical or representative of other cases*.” Yet later Stake states, “*Case study research is not sampling research. We do not study a case primarily to understand other cases*.” And then, “*A collective case study may be designed with more concern for representation but, again, the representation of a small sample is difficult to defend*.” (p. 4). Regarding how to select cases: 1) “*opportunity to learn is of primary importance*”; 2) “*selection by sampling of attributes*” is described, but is seen to be of secondary importance; 3) “*variety*” may be sought, and 4) accessibility and hospitability may also influence selection of cases (p 5-7). 2. Selecting data sources: “*Selection of data sources can be left too much to chance. The people who happen to be there when we happen to be there are not likely to be the best sources of data. The researcher should have a connoisseur’s appetite for the best persons, places, and occasions. ‘Best’ usually means those that best help us understand the case, whether typical or not*” (p. 56). The same four considerations for selecting cases are also described for selecting data sources. But, “*Each researcher is different; each has to work out methods that make him or her effective in understanding and portraying the case*” (p. 57). |
|  |  | 2005a | Cases;  Units within the case: people, places, events, subsections, groups, occasions, dimensions, domains | 1. Selecting cases: “*Instrumental and collective casework regularly requires cases to be chosen. Achieving greatest understanding of the critical phenomena depends on choosing the case well (Paton, 1990; Vaughan, 1992; Yin, 1989)… We want to generalize…, yet we realize that each of these cases, each sample of one, weakly represents the larger group of interest*” (p. 450). “*For qualitative fieldwork, we draw on a purposive sample, building in variety and acknowledging opportunities for intensive study… Even for collective case studies, selection by sampling of attributes should not be the highest priority. Balance and variety are important; opportunity to learn is often more important*” (p. 451). 2. Selecting data sources: “*The case is singular, but is has subsections (e.g., production, marketing, sales departments), groups (e.g., patients, nurses, administrators), occasions (e.g., work days, holidays, days near holidays), dimensions, and domains—many so well-populated that they need to be sampled*” (p. 449). “*They are cases within the case—embedded cases or mini-cases*” (p. 451). |
|  |  | 2005b | Cases: “people, activities, policies, strengths, problems, or relationships”  Units within the case: people, places, events | 1. “Deciding on issues for the quintain”: [Stake defines the quintain as a “*whole*” comprising “*the collection of these cases or…the phenomenon exhibited in those cases*,” and it is the “*official interest*” of a multicase study (p. vi). Issues are defined as the research question(s) that are common to all cases in the mulitcase study; they are “*important problems about which people disagree*,” and “*study of issues that cut across cases contributes to understanding the quintain*” (p. vi).] “*Deciding on issues for the quintain and the cases helps us define data sources and data-gathering activities… Examining these data, we will often revise our issues. New issues emerge. Case study work is often said to be ‘progressively focused’; that is, the organizing concepts may change a little or a lot as the study moves along*” (p. vi). A space for listing “*Issues*” is included on Stake’s worksheet titled, “Outline for gathering data on a case” (p. 5). 2. Selecting cases: “*When we choose, it is often better to pick the cases that most enhance our understanding than to pick the most typical cases. In fact, highly atypical cases can sometimes give the best insights into the quintain*” (p. vii). “*The selection process regularly begins with cases already at least partially identified*” (p. 22). Criteria for selecting cases include (as bullets): “*Is the case relevant to the quintain? Do the cases provide diversity across contexts? Do the cases provide good opportunities to learn about complexity and contexts?*” And secondarily, “*If other considerations are satisfied, cases will be selected because they represent the program or phenomenon*” (p. 23). |

### Table 9: Comparison of recommendations regarding ongoing sampling (e.g., theoretical sampling) across influential authors in phenomenology and case study

| **Tradition** | **Author** | **Ref.** | **Type(s) of ongoing sampling described and definition(s)** |
| --- | --- | --- | --- |
| Phenomenology | van Manen | 1997 | None |
|  | Giorgi | 2009 | None |
|  | Colaizzi | 1978 | None |
|  | Cohen | 2000 | 1. “Two-tier system of sampling”: The first tier of participants are interviewed according to a pre-planned protocol schedule, and the second tier consists of a more select group of participants who are followed more intensively. “*We chose this second tier of informants because…we found in their texts themes and refrains that had appeared in a number of the interviews with other informants.*” These themes were explored in more depth in subsequent interviews with the participants thus selected (p. 51). 2. To understand a participant’s “tradition”or “ways of talking about experience”: This is done by choosing people to interview who “*represent*” it: “*Exploring who shares a way of talking about a particular experience cannot be determined before the researcher enters the field*” (p. 54). It can be “*determined only through careful data collection*” (p. 55). |
| Case study | Yin | 2009 | None |
|  |  | 2011 | “Snowball sampling”: “*selecting new data collection units as an offshoot of existing ones—can be acceptable if the snowballing is purposeful, not done out of convenience. In the course of an interview you might learn of other persons who can be interviewed. The snowballing occurs when you follow such a lead and let those new ones result in identifying yet other possible interviewees*” (p. 89). Snowball sampling can be used to select “*participants or sources of data to be used in a study*” (p. 312). |
|  | Merriam | 2009 | 1. “Sampling within the case”: “*A sample within the case needs to be selected either before the data collection begins or while the data are being gathered (ongoing or theoretical sampling)*” (p. 81-82). 2. “Snowball/chain/network sampling”: “*perhaps the most common form of purposeful sampling. This strategy involves locating a few key participants who easily meet the criteria you have established for participation in the study. As you interview these early participants you ask each one to refer you to other participants*” (p.79). 3. “An ongoing sample selection process commonly referred to as theoretical sampling”: Part of the Glaser and Strauss’ 1967 definition of theoretical sampling is referenced, and then rephrased by Merriam: “*The researcher begins with an initial sample chosen for its obvious relevance to the research problem. The data lead the investigator to the next document to be read, the next person to be interviewed, and so on. It is an evolving process guided by the emerging theory. …As data are being collected and theoretical constructs begin to evolve, the researcher might also look for exceptions (negative-case selection) or variants (discrepant-case selection) to emerging findings*” (p.79-80). |
|  | Stake | 1995 | None |
|  |  | 2005a | None |
|  |  | 2005b | Choosing “issues” to define data sources (for multiple case study): [Stake defines “*issues*” as the research question(s) that are common to all cases in the mulitcase study; they are “*important problems about which people disagree*,” and “*study of issues that cut across cases contributes to understanding the quintain*” (p. vi).] “*Deciding on issues for the quintain and the cases helps us define data sources and data-gathering activities… Examining these data, we will often revise our issues. New issues emerge. Case study work is often said to be ‘progressively focused’; that is, the organizing concepts may change a little or a lot as the study moves along*” (p. vi). Referring to the developers of grounded theory: “*One can use a case study or multicase study as a step toward theory, as described by Barney Glaser and Anselm Strauss (1967; see also Ragin & Becker, 1992)*” (p. 8). |

### Table 10: Comparison of what is considered the unit of analysis

| **Tradition** | **Author** | **Ref.** | **Unit of analysis** |  |  |
| --- | --- | --- | --- | --- | --- |
| Grounded theory | Glaser | 1978 |  |  |  |
|  |  | 1992 |  |  |  |
|  |  | 1998 |  |  |  |
|  | Strauss | 1967 |  |  |  |
|  |  | 1973 |  |  |  |
|  |  | 1998 |  |  |  |
|  | Corbin | 2008 | “Question 3. ‘What is the focus of analysis, if not numbers?’ …For us, the unit of analysis is the *concept*. As explained in Chapter 7, the sampling procedures are designed to look at how concepts vary along a dimensional range, rather than measuring the distribution of persons along some dimension or a concept. Therefore, researchers collect data from places and/or persons and/or on things where they expect potential variations in that concept will be maximized.” (p. 316). |  |  |
|  | Charmaz | 2003 |  |  |  |
|  |  | 2006 |  |  |  |
|  | Clarke | 2005 |  |  |  |
| Phenomenology | van Manen | 1997 |  |  |  |
|  | Giorgi | 2009 |  |  |  |
|  | Colaizzi | 1978 |  |  |  |
|  | Cohen | 2000 |  |  |  |
| Case study | Yin | 2009 | “As a general guide, your tentative definition of the unit of analysis (and therefore the case) is related to the way you have defined your research questions” (p. 23). |  |  |
|  |  | 2011 |  |  |  |
|  | Merriam | 2009 | “I have concluded, however, that the single most defining characteristic of case study research lies in delimiting the object of study, the case. …case study is less of a methodological choice than ‘a choice of what is to be studied’ [citing Stake, 1995]… The unit of analysis, *not* the topic of investigation, characterizes a case study. For example, a study of how older adults learn to use computers would probably be a qualitative study but not a case study” (p. 40). |  |  |
|  | Stake | 1995 |  |  |  |
|  |  | 2005a |  |  |  |
|  |  | 2005b |  |  |  |

1. Gentles SJ, Charles C, Ploeg J, McKibbon KA**: Sampling in qualitative research: insights from an overview of the methods literatu**re*. The Qual Re*p 2015**,** 20(11):1772-1789.
